# The International Evidence-based Guidelines for the Assessment and Management of Polycystic Ovary Syndrome: An Exemplar of Person-centred Care

**DOI:** 10.17925/EE.2024.20.2.1

**Published:** 2024-08-16

**Authors:** Bharti Kalra, Nitin Kapoor, Atul Dhingra, Sanjay Kalra

**Affiliations:** 1. Department of Obstetrics and Gynaecology, Bharti Hospital, Karnal, Haryana, India; 2. Department of Endocrinology, Diabetes and Metabolism, Christian Medical College, Vellore, Tamil Nadu, India; 3. Non-Communicable Disease Unit, Baker Heart and Diabetes Institute, Melbourne, Victoria, Australia; 4. Department of Endocrinology, Sir Gangaram Bansal Hospital, Sri Ganganagar, Rajasthan, India; 5. Department of Endocrinology, Bharti Hospital, Karnal, Haryana, India; 6. University Center for Research & Development, Chandigarh University, Mohali, Punjab, India

**Keywords:** Family-centred care, obesity, person-centred care, polycystic ovary syndrome (PCOS), psychology, reproductive endocrinology, woman-centred care

## Abstract

In this opinion piece, we appraise the International Evidence-based Guideline for the Assessment and Management of Polycystic Ovary Syndrome 2023 from a person-centric perspective. We discuss how the authors balance evidence with empathy and offer excellence in clinical decision-making while ensuring the empowerment of the affected individual. We note how they skilfully use powerful words and phrases to capture the essence of person-centred care. Finally, we suggest how these guidelines can be strengthened and how they can be used to create a template for guidance on the management of other chronic disorders.

Polycystic ovary syndrome (PCOS) is a multifactorial, multifaceted syndrome that affects women across all ages from adolescence to post-menopause. It is reported to be the most common endocrinopathy in women of the reproductive age group.^1^ The nature of this syndrome is such that it requires support and intervention from multiple medical and allied specialties. The treatment depends on the chief complaints and concerns of the person involved. This calls for a person-centred, or a woman-centred, approach to the management of PCOS.

## Person-centred medicine

Person-centred care (PCC) is defined in various ways. The Institute of Medicine (Washington DC, USA) defines PCC as ‘care that is respectful of and responsive to individual patient preferences, needs and values’ and that ensures ‘that patient values guide all clinical decisions’.^2^ This definition keeps the person living with the disease at the centre of all decision-making. It neither means that we abdicate our responsibilities as healthcare providers, nor suggests that we abnegate our professional rights. South Asian researchers use the term ‘responsible PCC’, describing it as ‘that in which the physician or healthcare team take on the responsibility of ensuring that the person with diabetes is offered all relevant information, in an understandable manner, so that he or she can take part in a shared decision-making process, which offers the potential for achieving optimal therapeutic outcomes, without ignoring his or her biopsychosocial context’.^3^ We suggest an alliterative definition, which is appropriate not only for PCOS but also for all healthcare practices. We define PCC as care characterized by conscious compassion and compathic communication, placing the person’s complaints, concerns and challenges at the centre of conversation and clinical planning, while being cognizant of her convenience, comfort and cost-related issues.

## Evidence-based; empathy-laced

The 2023 PCOS guidelines build upon similar guidelines published in 2018.^4,5^ The authors of the current document reached out to professional societies, as well as consumer organizations, and engaged with medical experts, as well as women living with obesity. This inclusive strategy allowed the voices of all stakeholders, including women living with PCOS, to be incorporated.^4^ The guidance promotes care compassion and compathic communication; prioritizes the woman’s complaints, concerns and challenges; highlights the need for meaningful conversation and clinical planning and reinforces the necessity to optimize community and family support, while being mindful of comfort and cost.

## Person-centred thoughts and words

The authors humbly acknowledge that women are dissatisfied with the care that they receive for PCOS and work towards correcting this situation. The language in which the aim of the guideline is couched, ‘to ensure that women with PCOS receive optimal, evidence-based care…’ and ‘to support women and their health care providers…’, suggests a strong person-centred philosophy. This thought process is further strengthened by the emphasis on ‘partnership in care’, ‘empowerment of women’ and consideration of ‘personal characteristics, preference, culture and values’, ‘in addition to resource availability’. Empowerment is an important facet of PCOS management. This is reflected in the choice of verbiage: ‘shared decision-making’ to ‘support patient agency or ability to take independent actions’.^4^

## Screening and diagnosis

The five-section guidelines begin with a section on screening, diagnosis and risk stratification. This highlights the need for an age group-specific approach in PCOS evaluation. Adolescent PCOS presentation has subtle differences from that in adults: these are highlighted in the recommendations. Similarly, the need to care for women in the post-menopausal phase of life is underscored. Ethnic variations in the prevalence and presentation of PCOS are discussed as well.

Part of PCC is being aware of available resources, and practising parsimony in investigation as well as treatment. This aspect is dealt with by suggesting the use of appropriate assays and avoiding duplication of tests, such as anti-Müllerian hormone and ultrasonography.^4^

One example of strengthening screening of women for PCOS at a primary healthcare level is a screening tool for use in primary care settings. This tool consists of six questions, structured into three domains: menstrual/maternity, metabolic and ‘misfit masculinity’ (dermatological). The authors suggest that a positive answer to a question in any two domains should prompt suspicion of PCOS.^6^

## Psychological health

The equal focus on psychosocial and lifestyle aspects of PCOS is a praiseworthy attempt to bolster person-centricity.^4^ The allotment of a separate section to ‘prevalence, screening and management of psychological features’, as well as models of care, is commendable. While depression and anxiety are covered adequately, it may be worthwhile to discuss the concept of PCOS distress.^7^ Similar to diabetes distress, we define PCOS distress as a self-perceived inability to cope with the challenges and demands of living with PCOS.^8^ Although this does not necessarily warrant psychiatric labelling or consultation, it certainly requires counselling and support. This can be offered by healthcare providers, trained members of the lay public and other women living with PCOS.

## Lifestyle modification

The person-centred philosophy of the 2023 PCOS guidelines is evident in the section on lifestyle management. ‘Lifestyle management is a core focus’, and goals and priorities should be co-developed in partnership with women living with PCOS, while valuing ‘women’s individualized preferences’. Lifestyle management requires continuous or ‘ongoing assessment and support’. This is true for dietary intervention as well, where ‘a flexible, individual and co-developed approach to achieving nutritional goals’ is advocated. No single-diet composition or type/intensity of exercise is suggested. Rather, a ‘sustainable physical activity based on individual preferences and goals’ is recommended.^4^ Women living with PCOS should be encouraged to follow sensible and sustainable lifestyle choices that help optimize weight and other metabolic parameters. Balanced diets, with relatively less carbohydrate content, may help in weight management. Exercise regimens and routines should be chosen keeping medical, social and environmental factors in mind.

## Partners and family

The psychosexual function has also been discussed in the guidelines. It is important to involve the partner in sexual and all PCOS management. Having the partner as an active participant in PCOS care may improve adherence to, persistence with and satisfaction from therapy. In fact, the entire family should be engaged in PCOS care. The guidelines introduce the concept of a family-centric approach in screening and diagnosis, reminding readers that ‘fathers and brothers of women with PCOS may have an increased prevalence of metabolic syndrome, type 2 diabetes and hypertension’.^5^ More focus on family involvement may improve PCOS outcomes, as is evident from the diabetes world.^9^ Family members, including male relatives, may be offered screening services for blood pressure, glucose and weight at the same healthcare service venue, to promote family visits.

The reference to ‘the value of broader family engagement’ stresses that PCOS is not just an individual’s disease, but a family syndrome as well.^4^ Weight stigma, psychosexual issues and the possibility of a family history of metabolic syndrome are some ways in which the family is affected by PCOS. These can be handled by couple-centred or family-based counselling sessions, as well as social awareness campaigns.

## Societal involvement

The authors mention the need to involve society (‘public health actors should consider screening societal awareness and education on PCOS to reduce stigma and marginalization’), broaden health professional engagement (e.g. nurse practitioners) and consider ‘culturally appropriate resources and education on PCOS across the lifespan for families’. This underscores the impact that PCOS has on family and society and highlights the inclusive nature of the guidelines.^4^ One may wish to involve community leaders and religious leaders in a social marketing campaign to increase awareness of PCOS and enhance sensitivity towards women living with PCOS.

## Pharmacological management

A strong person-centric attitude is noticeable in the discussion on the management of non-fertility and fertility-related issues. ‘Shared decision-making’, including accurate information and reassurance, with a reminder that ‘individual’ characteristics, preferences and values must be elicited and considered and ‘understanding how individual adults and adolescents value treatment outcomes is essential’.^4^ Each woman will have a different perspective of success in therapy, and this perspective must be respected and responded to in an empathic and effective manner.

A person-centred approach to pharmacotherapy is clearly visible in the guidelines. Antidepressant medications are to be ‘considered in adults whose mental health disorders are clearly documented and persistent’ or ‘if suicidal symptoms are present’. While caution to ‘avoid inappropriate treatment [...] with antidepressants or anxiolytics’ is expressed, the fact that ‘not managing anxiety and depression may impact adherence’ is also highlighted.^4^

It is true that most countries have not approved medical therapy for ‘use specifically in PCOS’. In India, however, metformin and inositol are approved for use in this condition. This should be taken into account while planning therapy. The guidelines discuss the individual factors that will help decide whether to choose metformin or combined oral contraceptive pills in a particular woman. A similar approach is followed for choosing letrozole, clomiphene and metformin as ovulation-i nducing agents.

Inositol is the subject of discussion for both non-fertility and fertility-related targets. It ‘could be considered [...] based on individual preferences and values’, because of ‘limited harm [... and...] potential for improvement in metabolic measure’. However, women should ‘advise’ or inform their physician about this.^4^ Regulatory policies should also be discussed, and the current country-specific status should be shared with the patient. Greater research and focus on other forms of complementary and alternative medicine is needed to ensure the appropriate use of these types of therapy.

Mechanical laser and light therapies should be sought from ‘experienced and suitably qualified providers’. These help in ‘reducing facial hirsutism’ and are useful ‘for related depression, anxiety and quality of life’.^4^ Each country has its own regulations for the use of such treatments, and these should be respected and adhered to.

Assisted reproductive technology is covered in great detail, and person-centric factors that influence the choice of therapy are delineated. There is great emphasis on ‘counselling’ and on the fact that current evidence often does not allow the choice of a specific hormonal preparation.^4^

## Weighty issues

The use of the term ‘higher weight’, as opposed to overweight, reflects sensitivity to women’s needs. In fact, the guidelines encourage us ‘to seek permission’ before measuring weight/body mass index or ‘initiating a dialogue on the importance of weight and lifestyle on women’s health before pregnancy’. Caution is advised to avoid weight stigma while considering ‘the cultural, social and environmental determinants of healthcare’.^4^

Anti-obesity medications, including liraglutide, semaglutide and orlistat, are mentioned in the text. They ‘could be considered, in addition to active lifestyle intervention, for the management of higher weight in adults with PCOS as per general population guidelines’. ‘Gradual dose escalation’ and ‘concurrent effective contraception’ are advised to ensure the tolerability and safety of the drugs.^4^ It must be mentioned here that semaglutide is approved for use in adolescents living with obesity, above the age of 12 years, in the USA.^10^

## Information and education

The strong focus on providing ‘information-tailored education and resources’ in a ‘respectful and empathic manner’ is welcome. As rightfully stated, such offerings should be embedded in healthcare professional education as well. Equity-based models of care are necessary for the effective management of PCOS.^4^

Quaternary prevention (avoiding over-i nvestigations and over-treatment) and quinary prevention (preventing misinformation and promoting the right information) are recurring themes in the 2023 PCOS guidelines.^11,12^

As the authors state, ‘shared decision-making and self-empowerment are fundamental’ to PCOS care. ‘Integrated models of care should be co-designed, funded and evaluated’. These are the pillars of PCC, which respect, and are responsive to, the requirements of the person living with PCOS. The AskPCOS application and other e-health information resources, which accompany the guidelines, ensure that these words are converted into action.^13^

## Person-centred care, beyond polycystic ovary syndrome

The 2023 PCOS guidelines serve as a useful template for the creation of similar recommendations on the management of other chronic diseases. Endocrine diseases, such as diabetes and obesity, and gynaecological conditions, such as endometriosis, menopause and premenstrual syndrome, are examples that will benefit from such efforts. We hope that the 2023 PCOS guidelines will lead to improvement in healthcare delivery and outcomes, not only for women with PCOS, but also for people living with other chronic conditions. This is a trial of, and a tribute to, person-centred medicine.
